# The Interplay between S-Glutathionylation and Phosphorylation of Cardiac Troponin I and Myosin Binding Protein C in End-Stage Human Failing Hearts

**DOI:** 10.3390/antiox10071134

**Published:** 2021-07-16

**Authors:** Heidi Budde, Roua Hassoun, Melina Tangos, Saltanat Zhazykbayeva, Melissa Herwig, Marharyta Varatnitskaya, Marcel Sieme, Simin Delalat, Innas Sultana, Detmar Kolijn, Kamilla Gömöri, Muhammad Jarkas, Mária Lódi, Kornelia Jaquet, Árpád Kovács, Hans Georg Mannherz, Vasco Sequeira, Andreas Mügge, Lars I. Leichert, Samuel Sossalla, Nazha Hamdani

**Affiliations:** 1 Institut für Forschung und Lehre (IFL), Molecular and Experimental Cardiology, Ruhr University Bochum, 44801 Bochum, Germany; heidi.budde@rub.de (H.B.); Roua.Hassoun@rub.de (R.H.); melli-sw@web.de (M.T.); Saltanat.Zhazykbayeva@ruhr-uni-bochum.de (S.Z.); Melissa.herwig@ruhr-uni-bochum.de (M.H.); Marcel.Sieme@ruhr-uni-bochum.de (M.S.); Simin.Delalat@ruhr-uni-bochum.de (S.D.); innas.sultana@gmail.com (I.S.); detmarkolijn@gmail.com (D.K.); kamilla.gomori@gmail.com (K.G.); muhammadjarkas@icloud.com (M.J.); kornelia.jaquet@ruhr-uni-bochum.de (K.J.); kovacs.arpad@med.unideb.hu (Á.K.); Hans.Mannherz@ruhr-uni-bochum.de (H.G.M.); andreas.muegge@ruhr-uni-bochum.de (A.M.); 2 Department of Cardiology, St. Josef-Hospital and Bergmannsheil, Ruhr University Bochum, 44801 Bochum, Germany; 3 Institute of Biochemistry and Pathobiochemistry, Department of Microbial Biochemistry, Ruhr University Bochum, 44801 Bochum, Germany; Marharyta.Varatnitskaya@ruhr-uni-bochum.de (M.V.); Lars.Leichert@rub.de (L.I.L.); 4 Department of Neuroanatomy and Molecular Brain Research, Ruhr University Bochum, 44801 Bochum, Germany; maria.lodi@rub.de; 5 Department of Anatomy and Molecular Embryology, Ruhr University Bochum, 44801 Bochum, Germany; 6 Comprehensive Heart Failure Center (CHFC), University Clinic Würzburg, 97080 Würzburg, Germany; Sequeira_V@ukw.de; 7 Department of Internal Medicine II, University Medical Center Regensburg, 93042 Regensburg, Germany; samuel.sossalla@klinik.uni-regensburg.de; 8 Clinic for Cardiology & Pneumology, Georg-August University Goettingen, and DZHK (German Centre for Cardiovascular Research), Partner Site Goettingen, 37073 Goettingen, Germany

**Keywords:** myofilament proteins, oxidative stress, inflammation, phosphorylation, S-glutathionylation

## Abstract

Oxidative stress is defined as an imbalance between the antioxidant defense system and the production of reactive oxygen species (ROS). At low levels, ROS are involved in the regulation of redox signaling for cell protection. However, upon chronical increase in oxidative stress, cell damage occurs, due to protein, DNA and lipid oxidation. Here, we investigated the oxidative modifications of myofilament proteins, and their role in modulating cardiomyocyte function in end-stage human failing hearts. We found altered maximum Ca^2+^-activated tension and Ca^2+^ sensitivity of force production of skinned single cardiomyocytes in end-stage human failing hearts compared to non-failing hearts, which was corrected upon treatment with reduced glutathione enzyme. This was accompanied by the increased oxidation of troponin I and myosin binding protein C, and decreased levels of protein kinases A (PKA)- and C (PKC)-mediated phosphorylation of both proteins. The Ca^2+^ sensitivity and maximal tension correlated strongly with the myofilament oxidation levels, hypo-phosphorylation, and oxidative stress parameters that were measured in all the samples. Furthermore, we detected elevated titin-based myocardial stiffness in HF myocytes, which was reversed by PKA and reduced glutathione enzyme treatment. Finally, many oxidative stress and inflammation parameters were significantly elevated in failing hearts compared to non-failing hearts, and corrected upon treatment with the anti-oxidant GSH enzyme. Here, we provide evidence that the altered mechanical properties of failing human cardiomyocytes are partially due to phosphorylation, S-glutathionylation, and the interplay between the two post-translational modifications, which contribute to the development of heart failure.

## 1. Introduction

Muscular diseases are associated with changes to muscle proteins, and their functions are causally linked to imbalances in cellular antioxidant systems and free radicals, such as the production of reactive oxygen species (ROS), i.e., oxidative stress [1]. Muscular dystrophies, such as protein aggregate myopathies (PAM) [2], dysferlinopathy [3], Duchenne muscular dystrophy (DMD), and the mdx mouse model of DMD [4,5,6], all show increases in oxygen radical species. Although muscle proteins that are affected by oxidative modifications include various contractile and regulatory proteins of the sarcomere, the pathophysiological alterations in the myopathies resulting from oxidative stress are still poorly understood. A better characterization of the oxidative modifications that affect sarcomere proteins in myopathies, and their functional consequences, should provide us with a deeper understanding of the structural and functional remodelling events that take place in the affected muscles, and could be of considerable value when designing potential therapeutic interventions.

Oxidative stress and inflammation are also either a cause heart failure (HF), or a result of cardiovascular damage, and are thereby involved in the development of HF [7,8]. HF is a progressive multi-factorial disease, with a reduced output of the left ventricle and a sub-optimal supply of the tissues with oxygen and nutrients. Cardiac dysfunction is correlated with contractile dysfunction, cardiac remodeling, and inflammation processes that are caused by, or result in, oxidative stress. 

In cardiomyocytes, several intracellular sources of ROS/NOS are typically found, including mitochondria, nitric oxide synthases (NOS), xanthine oxidase (XO), NADPH oxidase (NOX), and cytochrome p450 [9,10,11]. Under physiological conditions, tissue oxidative balance is maintained by utilizing an antioxidant defense system that reduces harmful levels of ROS/NOS. The protective mechanisms that compete against ROS-mediated damage in all type of cells include different enzymatic pathways, for example, catalases and glutathione peroxidase, etc. In addition, a variety of antioxidant molecules, such as vitamins, glutathione, and the thiol groups of intracellular proteins, constitute an antioxidant buffer [12]. 

The oxidation of sarcomeric proteins, or proteins that are involved in calcium homeostasis, may affect contractile performance and signaling pathways in cardiomyocytes. Furthermore, their oxidation may induce structural remodeling processes and inflammation, by promoting the production of pro-inflammatory factors [1,13,14,15,16,17]. Elevated pro-inflammatory cytokines lead to inflammation, and, in turn, trigger the production of large amounts of ROS in neutrophils and macrophages [18,19]. Hence, these modifications modulate cardiomyocyte force production and stiffness in vitro [16,17,20,21,22].

Reversible S-glutathionylation is a protective mechanism of proteins against irreversible oxidation, by reduced glutathione (GSH) to a thiol group of the redox-sensitive cysteine residue. The balance between the reduced (GSH) and the oxidized form (GSSG) of glutathione is important for redox homeostasis [20]. Under oxidative stress, the GSH/GSSG ratio is reduced, while glutathionylation of myofilament proteins (excluding cardiac troponin T and myosin light chain 2, due to missing cysteine residues) alters the contractility of cardiomyocytes [23]. Lovelock et al. (2012) showed that, in hypertensive mice, S-glutathionylation of myosin binding protein C (MyBP-C), a sarcomeric protein that is responsible for the linkage of thick and thin filaments with the modulation of cross-bridge kinetics, correlated with enhanced myofilament Ca^2+^ sensitivity and diastolic dysfunction [24]. 

Not solely dependent on the oxidative modification to proteins, cardiac muscle function is additionally fine-tuned by phosphorylation and dephosphorylation of sarcomeric and calcium-handling proteins, including titin, MyBP-C, cardiac troponin I (cTnI), and phospholamban, respectively [25]. Under physiological conditions, cMyBP-C and cTnI (the inhibitory subunit of the troponin complex [26]) are the targets of PKA, upon ß-adrenergic stimulation. Their phosphorylation is responsible for myofilament Ca^2+^ desensitization, with subsequent acceleration of Ca^2+^ reuptake into the sarcoplasmic reticulum; PKA-dependent phosphorylation of phospholamban removes its inhibitory function on sarcoplasmic reticulum Ca^2+^ ATPase (SERCA) [26]. Contrasting in HF, the PKA-dependent phosphorylation of cTnI is reduced, while the PKC-mediated phosphorylation of cTnI is markedly increased, and associates with myofilament Ca^2+^ sensitization and impaired cellular relaxation [27]. Most importantly, protein modifications due to oxidative stress are likely involved in altered responses to phosphorylation signals [1,28]. Accordingly, studies have shown a crosstalk between S-glutathionylation and phosphorylation. Chakouri et al. (2018) showed, in isolated rat hearts, that under cardiac stress, MyBP-C S-glutathionylation was associated with reduced PKA-dependent phosphorylation of MyBP-C and cTnI, increased myofilament Ca^2+^ sensitivity, and reduced systolic and diastolic performance. Of note, S-glutathionylation of proteins prevents irreversible oxidation, but also plays a crucial role in cell signaling processes [29].

Here, we aim to understand how oxidative modifications of myofilament proteins affect the contractile function of single human cardiomyocytes. Therefore, we shed light on the molecular mechanisms driving the oxidative modification of sarcomeric proteins (especially cTnI, cMyBP-C and titin), and their role in contractile function modulation, as well as the interplay between the oxidation and phosphorylation of both the proteins.

## 2. Materials and Methods

### 2.1. Human Heart Tissues 

All procedures were performed according to the Declaration of Helsinki and were approved by the local ethics committee. Left ventricular (LV) myocardial tissue was obtained from explanted hearts (NYHA class III or IV; *n* = 10; average age, 57 years) as well as from LV tissue from controls (non-failing human hearts) (*n* = 10; male; average age, 40 years). Non-failing cardiac LV tissue was obtained from donor human hearts for which no suitable transplant recipient was found. The donors had no history of cardiac disease, a normal ECG and normal ventricular function on echocardiography performed within 24 h prior to heart explantation. Tissues were restored in cardioplegic solution deep frozen in liquid nitrogen and then stored at −80 °C until use. Medication included angiotensin-converting enzyme inhibitors, angiotensin-II receptor, β-blockers, digoxin, or anti-arrhythmic agents. Samples were obtained after informed consent and with approval of the local ethics committee (20-6976-BR and 20-6976-1-BR). 

### 2.2. Force Measurements in Single, Skinned Cardiomyocytes

Force measurements were performed on single isolated skinned cardiomyocytes (*n* = 16–20/4 heart/group) as previously described [30,31]. LV samples were defrosted in relaxing solution and mechanically demembranated and incubated for 5 min in relaxing solution administrated with 0.5% Triton X-100 (all from Sigma-Aldrich, St. Louis, MO, USA). The cell suspension was washed 5 times in relaxing solution. Single cardiomyocytes were attached between a force transducer and the piezoelectric motor.

Passive force of the cardiomyocyte (F_passive_) was measured in relaxing within a sarcomere length (SL) range between 1.8 and 2.3 μm. Force values were normalized to cardiomyocyte cross-sectional area. Cardiomyocyte (*n* = 16–20/4 heart/group) F_passive_ was subsequently measured within an SL range between 1.8 and 2.3 μm as described above. The cardiomyocyte was then adjusted to 2.2 μm SL and exposed to a series of solutions with different calcium concentrations ranging from pCa 9.0 (relaxing) to pCa 4.5 (maximal activation) to obtain the force–pCa relation. Mean values on relative force (and tension) vs. pCa diagrams were fit with the “Hill” equation, resulting in a sigmoidal curve.

The forces were recorded at baseline and after anti-oxidant treatment, reduced glutathione (GSH) 30 min (10 mM; Sigma-Aldrich, St. Louis, MO, USA), oxidant (oxidized glutathione: GSSG) 30 min, protein kinase A (PKA, 100 U/mL; batch-12K7495; Sigma-Aldrich, St. Louis, MO, USA) and 6 mmol/L dithiothreitol (DTT; MP Biochemicals, Irvine, CA, USA) or in the presence of recombinant human PKCα (PKCα, 10 μg/mL, Sigma-Aldrich, St. Louis, MO, USA, P1782; batch 93K0330) activated in Ca^2+^-containing solution (pCa 5.9) including 6 mmol/L DTT, 1 μmol/L phorbol-12-myristate 13-acetate; Sigma-Aldrich, St. Louis, MO, USA), and 50 nmol/L calyculin A (Sigma-Aldrich, St. Louis, MO, USA). All incubations were performed for 30 min to 40 min in relaxing solution.

### 2.3. Myocardial Protein Kinase a (PKA) and Myocardial Protein Kinase C (PKC) Activity

For the measurements of PKA and PKC activities, a non-radioactive PKA and PKC kinase activity assay kit (Enzo Life Science, Farmingdale, NY, USA) was used. LV tissue samples were homogenized in cell lysis buffer as described before [31,32]. After centrifugation at 13,000 rpm for 30 min, the supernatants with equal amounts of total protein (30 ng/μL protein aliquots) were assayed according to manufacturer’s instructions, then the PKA and PKC substrates were added to the microliter plate to the appropriate wells. The kinase reaction was initiated after adding the ATP, then samples were incubated at 30 °C for 90 min. The phosphorylated peptide substrates, which arise during the enzyme reaction, were detected by a phospho-specific antibody, which in turn is bound to a peroxidase-conjugated secondary antibody (anti-rabbit IgG:HRP). The colorimetrical assay was developed with tetramethylbenzidine and measured in a microplate reader at 450 nm. The specific activity of PKA and PKC was expressed as ng/μL and μg/μL, respectively.

### 2.4. Myocardial Protein Kinase G (PKG) Activity

PKG activity was analyzed using a radioactive assay. LV tissue samples were homogenized in a buffer and centrifugated for 5 min as described before [18,32,33]. Reaction solutions containing 40 mM Tris-HCl (pH 7.4), 20 mM Mg (CH_3_COO)_2_, 0.2 mM [^32^P] adenosine triphosphate (ATP) (500–1000 cpm pM–1; Amersham PLC, Little Chalfont, United Kingdom), 113 mg/mL heptapeptide (RKRSRAE), and 3 μM cGMP (both from Promega Corp., Madison, WI, United States), and a highly specific inhibitor of cyclic adenosine monophosphate-dependent protein kinase (5–24; Calbiochem, San Diego, CA, United States) were incubated at 30 °C for 10 min. The reaction was subsequently terminated by adding 70 μL onto Whatman P-81 filters (MACHEREY-NAGEL). The samples were incubated and washed with 75 mM H_3_PO_4_ for 5 min to remove unbound ATP. The filters were washed with 100% ethanol and air-dried. For the measurement of PKG activity, a Wallac 1409 liquid scintillation counter (Hidex Oy, Turku, Finland) was used. Here, the specific activity of PKG was analyzed by quantification of the incorporated pM of ^32^P into the substrate (pM/min/mg protein).

### 2.5. Protein Analysis by Western Blot

To separate titin isoforms, polyacrylamide gel electrophoresis (PAGE) was performed as previously described [31]. Briefly, LV tissue samples (*n* = 8–10/group) were solubilized in a modified Laemmli buffer (50 mM Tris-HCl, pH 6.8, 8 M urea, 2 M thiourea, 3% SDS, 0.03% ServaBlue, 10% glycerol, 75 mM DTT). Samples were heated for 3 min at 96 °C and centrifuged. Samples were applied in duplicates, at concentrations within the linear range of the detection system between 20 μg and 25 μg dry weight; checked by spectroscopic methods) and separated by agarose-strengthened 2% SDS-PAGE. Gels were run at 1.8 mA constant current for 14–16 h. For the small proteins expression and phosphorylation, 8 to 15% SDS-PAGE was used. Subsequently, the proteins were transferred to polyvinylidene difluoride (PVDF) membranes (Immobilon-P 0.45 µm; Merck Millipore, Burlington, MA, USA) and pre-incubated with 3% bovine serum albumin in Tween Tris-buffered saline (TTBS; containing: 10 mM Tris-HCl; pH 7.6; 75 mM NaCl; 0.1% Tween; all from Sigma-Aldrich, St. Louis, MO, USA) for 1 h at room temperature. Then, blots were incubated overnight at 4 °C with the appropriate primary antibodies (see list of antibodies below).

To study PKA, PKC, PKG and cTnI oxidation, lysates were prepared in lysis buffer containing snap-frozen human heart tissue. For non-reducing SDS-PAGE, 100 mM N-3 ethylmaleinimide (Sigma, St. Louis, MO, USA) instead of DTT was added to the lysis buffer to prevent artificial thiol oxidation during sample preparation. 

Samples were applied at a concentration that was within the linear range of the detection system, as follows: 20–25 μg (dry weight) for titin blots, 5 μg for anti-phospho-cTnI (S23/S24) and 25 μg for other proteins blots. After separation, proteins were transferred to PVDF membranes. Blots were pre-incubated with 3% bovine serum albumin in Tween Tris-buffered saline (TTBS) for 1 h at room temperature. Then, blots were incubated overnight at 4 °C with the primary antibodies against the respective (phospho)protein; see the list of all antibodies used (Table 1). For S-glutathionylation, we used anti-glutathione from Abcam, Cambridge, UK (ab195341:1000). The primary antibody binding was visualized using a secondary horseradish peroxidase-labeled, goat anti-rabbit/mouse antibody (dilution 1:10,000; OriGene Technologies GmbH and Jackson ImmunoResearch Europe Ltd., respectively) and enhanced chemiluminescence (ECL Western blotting detection, Amersham Biosciences, Little Chalfont, UK). Staining was visualized using the Bio-Rad image reader (460 nm/605 nm Ex/Em; 2 s illumination) and signals were analyzed with Multi Gauge V3.2. All signals of small proteins were normalized to GAPDH (dilution 1:1000; Sigma, St. Louis, MO, USA), stained on the same blots. For titin analysis, PVDF stains were saved for comparison of protein load. 

The following primary antibodies were used for Western blots and imaging:

### 2.6. Oxidation Detection (OxICAT)

In the OxICAT method, reduced cysteines are specifically differentially labeled with ^12^C ICAT and oxidized cysteines after their reduction with ^13^C ICAT. The ICAT reagent also contains an affinity tag, which facilitates isolation and concentration of tagged peptides before MS analysis. OxICAT labeling was performed, as follows.

The starting materials for the OxICAT method (Cleavable ICAT^®^ reagent kit for protein labeling, (Sciex, Framingham, USA), not commercially available at present) consisted of frozen human heart tissue material with a size of approximately 2 × 2 mm stored in liquid nitrogen. The following steps were performed under anaerobic conditions to protect the tissue from disease-unrelated oxidation events. Before starting, the DAB buffer (6 M urea, 10 mM EDTA, 200 mM Tris/HCl with pH 8.5 and 0.5% SDS) was prepared and incubated in an anaerobic environment for 4 h to remove all oxygen from the solution. The tissue samples were placed in the anaerobic chamber, and 80 µL DAB buffer with 20 µL acetonitrile (ACN) were added to homogenize the tissue. For each tissue homogenate, one vial of the light ICAT reagent was used and samples (*n* = 5 per group) were incubated for 2 h at 37 °C and 1300 rpm in the dark. The next steps were performed under normal aerobic conditions. The reaction was stopped by adding 400 µL of ice-cold acetone and proteins were precipitated over night at −20 °C. The next day, samples were centrifuged at 13,000× *g* for 30 min at 4 °C. The supernatant was then discarded and another 400 µL of ice-cold acetone was added for washing. After repeating the centrifugation step, the remaining acetone was removed by evaporation at 25 °C until samples were completely dry. The protein pellets were resuspended in 80 µL of DAB buffer. For reducing oxidized cysteins, 2 µL of 50 mM Tris(2-carboxyethyl)phosphine hydrochloride (TCEP) was added to the samples and incubated at 37 °C for 10 min at 300 rpm. Afterwards, 20 µL of ACN was combined with one vial of heavy ICAT. Each sample was filled into one vial and incubated for 2 h at 37 °C and 1.300 rpm. Again, proteins were precipitated via 400 µL of ice-cold acetone for 4 hat −20 °C before being centrifuged and washed twice. After the last washing step, the acetone was evaporated at 25 °C. The dried protein pellets were mixed with 80 µL of denaturing buffer (50 mM Tris pH 8.5 and 0.1% SDS) and 20 µL of ACN. The samples were digested with trypsin over night at 37 °C. The following cation exchange and affinity chromatography were performed according to the manufacturer’s protocol and components of the kit. During these steps, all unlabeled and non-cysteine-containing peptides were removed. For mass spectrometry analysis, the flow-through containing the digested and labeled peptides was dried in a vacuum centrifuge. To remove the biotin tag, the two cleavage reagents A and B (95:5) were mixed and 90 µL was added to each sample. After vortexing, the mixture was incubated at 37 °C for 2 h at 300 rpm. The samples were concentrated in a vacuum centrifuge for 20 min. Then, 4 µL of sample was combined with 46 µL of a 0.1% trifluoroacetic acid (TFA) solution of which 30 µL was filled into HPLC tubes. Afterwards, the peptide samples were analyzed using mass spectrometry. 

### 2.7. Mass Spectrometry Data Analysis

ICAT-labeled peptides were analyzed by LC-MS/MS and evaluated using MaxQuant software (version 1.6.17.0), including the integrated search algorithm, Andromeda. The search was performed against the human proteome from the Uniprot database (taxonomy ID 9606, October 2019). For the Andromeda search, two miscleavages were allowed, oxidation (M) was chosen as variable modification. The parent ion mass tolerance was set to 10 ppm, and the fragment ion mass tolerance set to 0.5 Da. Identified peptides and their respective ICAT quantification were assessed using the “peptides.txt” MaxQuant output file and processed using Microsoft Excel. The percentage oxidation change was also calculated for the individual peptides for each sample. The heavy ICAT signal was divided by the total intensity (heavy (H) intensity + light (L) intensity) for that peptide, to determine the percentage of the intensity due to oxidation for each peptide, and the formula is as follows:

(heavy (H) signal/total signal) * 100 = percentage of intensity from oxidation = % oxidation.

In a next step, invalid values and contaminants were filtered out. The cTnI and cMyBP-C peptides were assigned to the associated protein domains and the exact position of the cysteines was determined using the canonical human protein sequence from the UniProt database (Uniprot IDs P19429 and Q14896, respectively).

### 2.8. Duolink In Situ PLA (Proximity Ligation Assay (PLA)) Technology

The in situ Duolink^®^ PLA technique was used to visualize protein–protein interaction in fixed human LV tissue with confocal microscopy. According to the Duolink^®^ PLA protocol the Duolink^®^ blocking solution, the Duolink^®^ antibody diluent, the Duolink^®^ PLUS and MINUS probes, and the Duolink^®^ buffer were mixed before use. 

Deparaffinated LV tissue was stained with wheat germ agglutinin (WGA) 5 µg/mL in PBS for 5 min, washed with PBS twice, permeabilized with 1% Triton X-100 in PBS for 5 min and washed with PBS again.

For the Duolink^®^ PLA technique the slides with the fixed, WGA-stained and permeabilized heart samples were incubated with 1 drop of the blocking solution in a pre-heated humidity chamber for 60 min at 37 °C. After blocking, the primary antibody was diluted in the Duolink^®^ antibody diluent and applied to the slides at 4 °C in the humidity chamber overnight. Antibodies used for the staining are listed at the end of this paragraph. For the interaction study, the two primary antibodies were used from two different host species. To remove unbound antibodies, the slides were washed 2× for 5 min with 1× wash buffer A at room temperature. For the detection of the primary antibody one PLA PLUS probe directed against rabbit antibodies and one PLA MINUS probe directed against mice antibodies were used. For this step the PLA probes (PLUS and MINUS) were diluted 1:5 in the Duolink^®^ antibody diluent in a reaction volume of 40 µL for one tissue sample and incubated in a pre-heated humidity chamber for 1 h at 37 °C. Following the incubation of the proximity probes, the slides were washed 2× for 5 min in wash buffer A at room temperature and the ligase was added in a 1:40 dilution in the prediluted 1× ligation buffer in the reaction volume of 40 µL for one sample. The ligation reaction in which the connector oligos hybridize to the PLA probes to create a circular DNA template was performed in the pre-heated humidity chamber for 30 min at 37 °C. Two washing steps were performed for 5 min with wash buffer A at room temperature after the ligation reaction, to remove unbound PLA probes and enzymes. For the amplification of the circular DNA, the polymerase was added to the prediluted 1× light-sensitive amplification buffer in a 1:80 dilution in a reaction volume of 40 µL. The amplification reaction was performed for 100 min in a pre-heated humidity chamber at 37 °C and protected from light. Following the amplification reaction, the slides were washed 2× for 10 min with prediluted 1× wash buffer B and afterwards washed with 0.01× wash buffer B for 1 min at room temperature. To mount the slides with a coverslip, the wash buffer was tapped off, a minimal amount of Duolink ^®^ PLA mounting medium with DAPI was added and the edges were sealed with nail polish. We used imaging confocal laser scanning microscopy (Nikon Eclipse Ti-E Inverted Microscope System; Nikon Instruments, Nikon Corp, Shinagawa, Tokyo, Japan). The slides could be stored in the dark at −20 °C for up to 6 months.

### 2.9. Immunofluorescence Imaging

The frozen LV tissue slides were air-dried for 10 min, rehydrated with PBS and fixed with 4% paraformaldehyde in phosphate-buffered saline (PBS) for 5 min. Then the slides were washed three times for 5 min with PBS. Afterwards the tissue was incubated with wheat germ agglutinin (WGA) for 5 min and washed three times for 5 min with PBS. Next, the slides were blocked with 5% BSA/PBS and 0.1% Triton for 1 h at room temperature. After washing 3× times with PBS, the primary antibody was diluted in 5% BSA/PBS and incubated overnight at 4 °C. Subsequently, the slides were incubated with the secondary antibody diluted in 5% BSA/PBS overnight at 4 °C. The slides were then washed again three times with PBS, covered with ultrathin glass coverslips and sealed with Mowiol mounting medium and ultrathin glass coverslips overnight at 4 °C. For the imaging, confocal laser scanning microscopy (cLSM) (Nikon Eclipse Ti-E inverted Microscope System, Nikon Instruments, Nicon Corp, Shinagawa, Tokyo, Japan) was used.

### 2.10. Quantification of Oxidative Stress and Inflammation Parameters of End-Stage Human Heart Failure Tissue 

Oxidative stress and inflammatory parameters of end-stage human failing hearts (*n* = 8–10 samples) were measured before and after treatment with GSH using colorimetric assay and enzyme-linked immunosorbent assay kits (ELISA). The following kits were used for the measurements: lipid peroxidation (malondialdehyde) assay kit (ab118970; Abcam, Cambridge, UK), 3-nitrotyrosine ELISA kit (ab116691; Abcam, Cambridge, UK), interleukin-6 (IL-6) ELISA kit (ab100772; Abcam, Cambridge, UK); intercellular cell adhesion molecule-1 (ICAM1) ELISA kit (ERICAM1; Thermo Fisher Scientific, Darmstadt, Germany), vascular cell adhesion molecule-1 (VCAM1) ELISA kit (KHT0601; Thermo Fisher Scientific, Darmstadt, Germany); and tumor necrosis factor alpha (TNFα) ELISA kit (ab108913; Abcam, Cambridge, UK). To analyze the hydrogen peroxide (H_2_O_2_) level in LV tissue homogenates, a colorimetric assay was used and the H_2_O_2_ formation was measured at 540 nm. For the calculation, a standard curve with a known concentration of H_2_O_2_ was used.

### 2.11. Quantification of Nitric Oxide (NO) Level of End-Stage Human Failing Hearts 

The NO concentration was measured before and after treatment with GSH using a colorimetric assay kit and by analyzing the ratio of total nitrate/nitrite (BioVision Inc., Milpitas, CA, USA). LV tissue samples were treated with trichloroacetic acid (8 g in 80 mL acetone; Sigma-Aldrich, St. Louis, MO, USA) and washed with 1 mL 0.2% DTT. Afterwards, the tissue samples were homogenized in 1% SDS sample buffer (tri-distilled water: 8.47 mL; glycerol: 2.1 mL; 10% SDS: 1.4 mL; 0.5 M Tris-HCl (pH 6.8): 1.75 mL; bromophenol blue: 0.28 mL; DTT: 32.4 mg; all from Sigma-Aldrich, St. Louis, MO, USA ) and underwent sonication. After centrifugation at 14,000× *g* for 15 min at 5 °C, the supernatants containing equal amounts of total protein were analyzed for NO concentration. The quantification of this assay is based on the NO production by providing a measure of total nitrate/nitrite. Thereby, nitrate was converted to nitrite using nitrate reductase. Then, Griess reagents converted nitrite to an azo chromophore reflecting NO concentration in the tissue samples. The nitrite levels were measured independently from nitrate by omitting the conversion of nitrate to nitrite using nitrate reductase. The absorbance was measured at 540 nm using a plate reader. To calculate the nitrite and nitrate concentrations, a standard curve was used.

### 2.12. Statistical Analysis

Data are given as the mean values ± SEM. Student’s *t*-test of two groups of parametric data was used for statistical analysis, while for non-parametric data Mann–Whitney U test was used. For more than two groups one-way ANOVA was used for the analysis of parametric data. *p*-values were corrected for multiple comparisons by the Tukey method. For analysis of proportions, Fisher’s exact test was used. Analysis was performed using GraphPad Prism 5–8. *p*-values are two-sided and considered statistically significant if *p* < 0.05.

## 3. Results

### 3.1. Altered Maximum Ca^2+^-Activated Tension and Ca^2+^ Sensitivity of HF Demembranated Cardiomyocytes 

**Figure 1:**
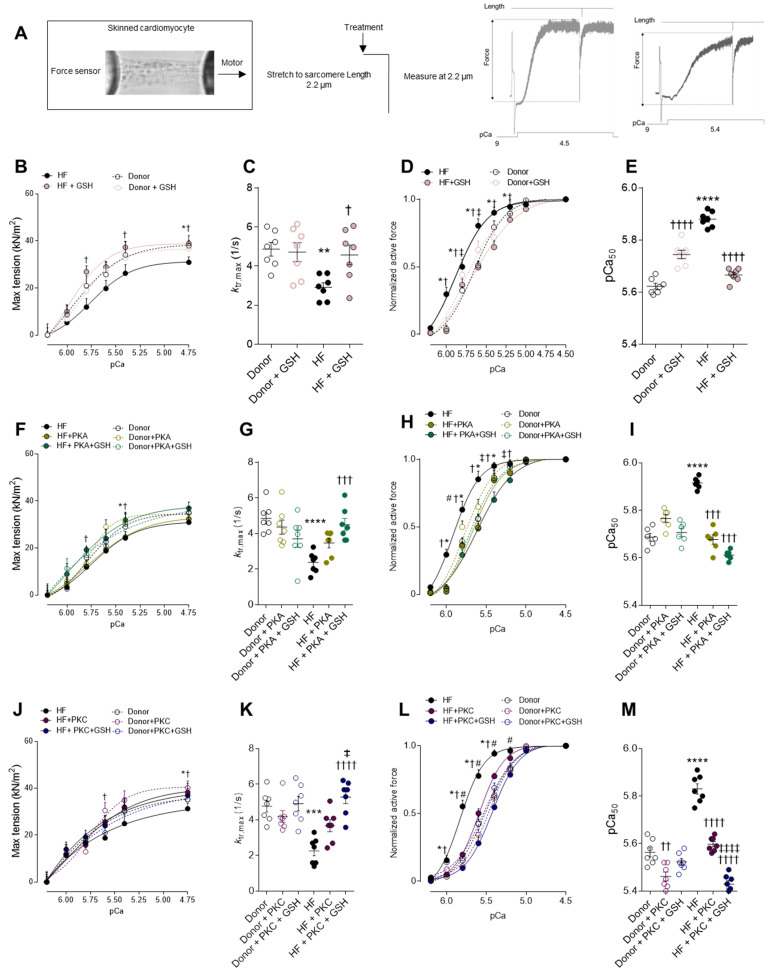
Cardiomyocyte max tension and calcium sensitivity. (**A**) Representative image of skinned cardiomyocytes and stretch protocol. (**B**) Maximum tension of donors and HF cardiomyocytes before and after reduced glutathione (GSH) treatment at different calcium concentrations. (**C**) Bars indicate ktr at saturating [Ca^2+^] (at pCa 4.5; ktr, max) before and after GSH treatment. (**D**) Calcium sensitivity of donors and HF cardiomyocytes with and without GSH treatment at different calcium concentrations. (**E**) pCa value for the half-maximal Ca^2+^-induced contraction before and after GSH. (**F**) Maximum tension of donors and HF cardiomyocytes with and without protein kinase A (PKA) treatment, and after incubations with both PKA and GSH at different calcium concentrations. (**G**) Bars indicate ktr at saturating [Ca^2+^] (at pCa 4.5; ktr, max) before and after PKA treatment. (**H**) Calcium sensitivity of donors and HF cardiomyocytes before and after PKA treatment at different calcium concentrations. (**I**) pCa value for the half-maximal Ca^2+^-induced contraction before and after GSH. (**J**) Maximum tension of donors and HF cardiomyocytes with and without protein kinase C (PKC) treatment and after incubations with both PKC and GSH at different calcium concentrations. (**K**) Bars indicate ktr at saturating [Ca^2+^] (at pCa 4.5; ktr, max) before and after PKC treatment. (**L**) Calcium sensitivity of donors and HF cardiomyocytes before and after PKC treatment at different calcium concentrations. (**M**) pCa value for the half-maximal Ca^2+^-induced contraction before and after GSH. Data are shown as mean ± SEM, (*n* = 16–20/4 cardiomyocytes/heart). * *p* < 0.05/** *p* < 0.01/*** *p* < 0.001/**** *p* < 0.0001 donor vs. HF, † *p* < 0.05/†† *p* < 0.01/††† *p* < 0.001/†††† *p* < 0.0001 HF before vs. after GSH or PKA, ‡ *p* < 0.05 HF before vs. after GSH + PKA or +PKC, and # *p* < 0.05 donor before vs. after PKA by one-way ANOVA. P-values were corrected for multiple comparisons by the Tukey method.

The maximum Ca^2+^-activated tension of single demembranated cardiomyocytes from end-stage failing human hearts was found to be reduced when compared to donors (Figure 1A,B). As myocardial oxidative stress is a potential trigger for HF-associated myofilament dysfunction, rescue experiments were attempted upon incubation with reduced glutathione (GSH) (Figure 1B). The maximum Ca^2+^-activated tension significantly increased in human HF cardiomyocytes after GSH treatment, whereas the effect was unchanged in the donor myocytes (Figure 1B). Furthermore, we investigated the mechanical ability of GSH in restoring the reduced maximal tension following PKA and PKC treatment. The incubations with either PKA (Figure 1F) or PKC (Figure 1J) mildly increased the maximum Ca^2+^-activated tension in HF myocytes, suggesting that the contribution of the protein’s oxidation to myofilament function is much larger (Figure 1B,F,J). In addition, the cross-bridge cycling kinetics (ktr) were significantly lower in HF compared to the donor, which is indicative of the slowing of actin–myosin turnover at saturating [Ca^2+^], and this was reversible upon treatment with GSH, but no effect was observed after PKA and/or PKC treatment (Figure 1C,G,K).

The force–pCa relationship of single demembranated cardiomyocytes revealed significantly higher myofilament Ca^2+^ sensitivity (pCa_50_) in HF compared to the donors (Figure 1D). The treatment with GSH resulted in a rightward shift in the normalized force–pCa curves of HF myocytes, which is indicative of Ca^2+^ desensitization following GSH treatment (Figure 1D). The Ca^2+^ sensitivity of the contractile apparatus was significantly reduced in HF cardiomyocytes upon incubation with PKA, whereas in the donor’s myocytes, this effect was blunted (Figure 1H). Similar results were obtained upon the incubation of HF myocytes with PKC (Figure 1L). Notably, the Ca^2+^ sensitivity was further reduced upon incubations with GSH, following either PKA or PKC (Figure 1F,L), suggesting that PKA- and PKC-mediated phosphorylation of cTnI are not sufficient to fully reverse the high Ca^2+^ sensitivity of end-stage human failing cardiomyocytes. The pCa value for the half-maximal Ca^2+^-induced contraction (pCa_50_) was significantly higher in the HF group compared to the donor group; this increase was reversed upon treatment with GSH, as well as after treatment with PKA and/or PKC (Figure 1E,I,M), indicating a full recovery of myofilament function using the combination of anti-oxidant and kinases.

### 3.2. Alterations in Activity and Oxidation of Major Protein Kinases and the Effect on Phosphorylation of Ctni and Cmybp-C in End-Stage Human Failing Hearts

**Figure 2:**
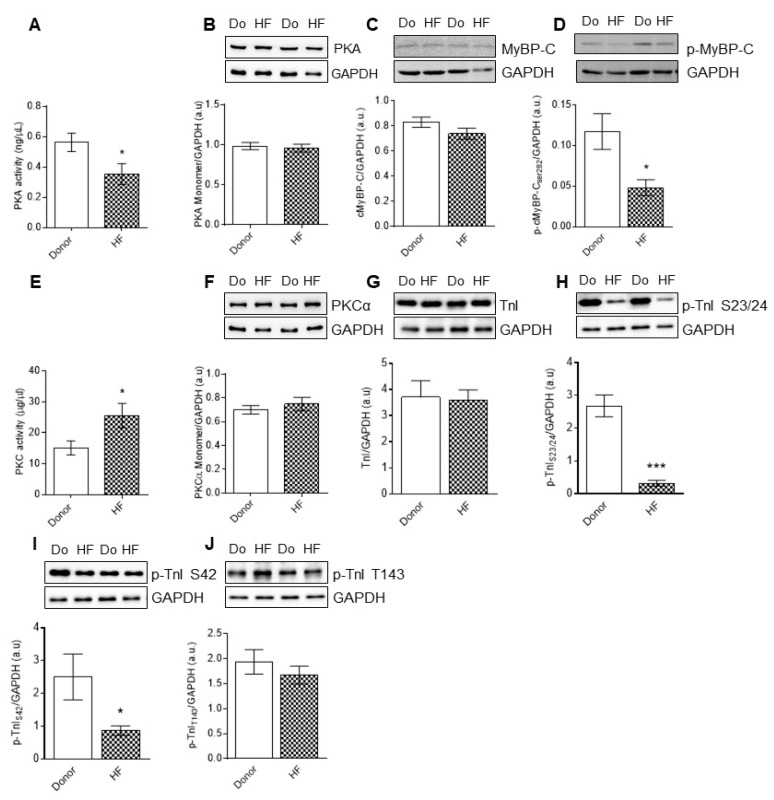
PKA and PKC activities, and cMyBP-C and cTnI phosphorylation of myocardial biopsies of human HF patients. (**A**) Protein kinase A (PKA) activity. (**B**) PKA monomer levels. (**C**) Cardiac myosin binding protein-C (cMyBP-C) levels. (**D**) Total cMyBP-C phosphorylation. (**E**) Protein kinase C (PKC) activity. (**F**) PKCα monomer levels. (**G**) Cardiac troponin I (cTnI) levels. (**H**) Phospho(P)-site-specific within cTnI at Ser23/24. (**I**) P-site-specific within cTnI at Ser42. (**J**) P-site-specific within cTnI at Thr 143. Data are shown as mean ± SEM. * *p* < 0.05/*** *p* < 0.001 donor vs. HF by Student *t*-test (*n* = 8–10, hearts per group).

The activity of PKA was reduced (Figure 2A), whereas PKC activity was significantly elevated in the end stage of failing heart samples (Figure 2E). We wondered if altered PKA and PKC activity is caused by target oxidation of the kinases, and therefore checked their oxidation using mass spectrometry and Western blotting. Single monomeric bands are observed for PKA and PKC (Figure 2B,F), implying that both the kinases are potentially non-oxidized in HF, and hence, the changes in kinase activity are independent of kinase oxidation. 

The protein expression levels of cTnI and cMyBP-C are unchanged in HF compared to the donors (Figure 2C,G); however, hypo-phosphorylation of cMyBP-C at S282 mouse canonical sequence and S284 human canonical sequence was observed in HF biopsies compared to the donors (Figure 2D). We found PKA-mediated phosphorylation sites of cTnI at sites S23/S24 and S43 mouse canonical sequence, which correspond to S42 (human canonical sequence), to be significantly reduced in HF compared to the donors (Figure 2H,I), whereas PKC-mediated cTnI phosphorylation at T143 was unchanged (Figure 2J). 

### 3.3. cTnI and cMyBP-C Glutathionylation in End-Stage Human Failing Hearts

**Figure 3:**
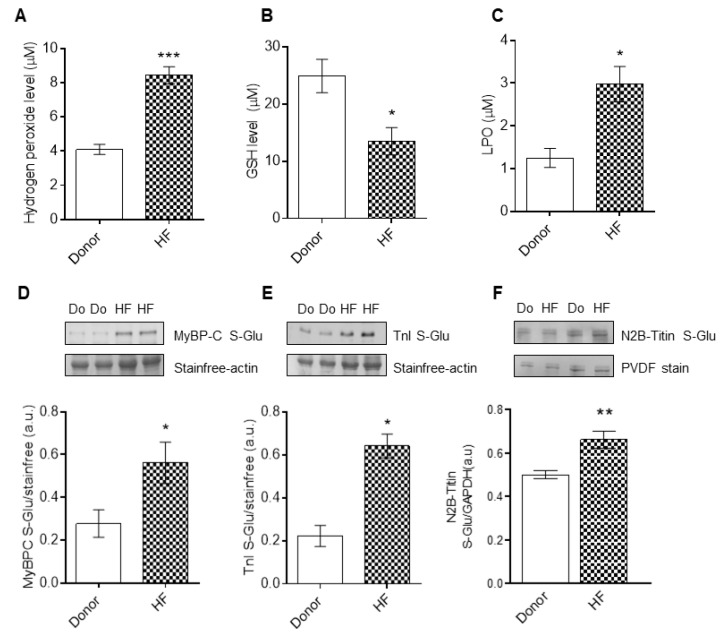
Myocardial oxidative stress parameters, and S-glutathionylation of cTnI and cMyBPC. (**A**) Hydrogen peroxide (H_2_O_2_) concentration levels. (**B**) Reduced glutathione (GSH) concentration levels. (**C**) Lipid peroxide (LPO) concentration levels. (**D**) S-glutathionylation of cMyBPC. (**E**) S-glutathionylation of cTnI. (**F**) S-glutathionylation of N2B-titin. Data are shown as mean ± SEM. * *p* < 0.05/** *p* < 0.01/*** *p* < 0.001 donor vs. HF by Student *t*-test (*n* = 8–10, hearts per group).

In the HF samples, we found increased concentration levels of hydrogen peroxide (H_2_O_2_) and lipid peroxide (LPO) (Figure 3A,C), but decreased concentration levels of reduced glutathione (Figure 3B). Furthermore, as cMyBP-C and cTnI post-translational modifications contribute to the modulation of maximum Ca^2+^-activated tension and Ca^2+^ sensitivity of cardiomyocytes, we turned our attention towards the putative oxidative modulation of cTnI and cMyBP-C. Western blot analysis revealed glutathionylation of cMyBP-C and cTnI in the end-stage failing hearts compared to the donors (Figure 3D,E). Along the lines, we found titin to additionally be S-glutathionylated in HF compared to the donors (Figure 3F). 

### 3.4. Identification of Oxidized Cysteins in Cmybp-C and Ctni Using Oxicat Together with Mass Spectrometry

We used the OxICAT method, coupled with mass spectrometry (MS), to assess the potential oxidative modifications of cysteines in muscle proteins [16]. OxICAT involves the use of a cysteine-specific isotope-coded affinity tag (ICAT) reagent to differentially label the oxidized and reduced cysteines, which can be detected via MS. By thoroughly labeling all the reduced cysteine residue (Cys) in the heart samples, and subsequently labeling the remaining Cys residues that are in an oxidized state in the heart tissues, we were able to reduce the artificial oxidation and quantify changes in redox state in the HF hearts and donor hearts in a reliable way. 

**Figure 4:**
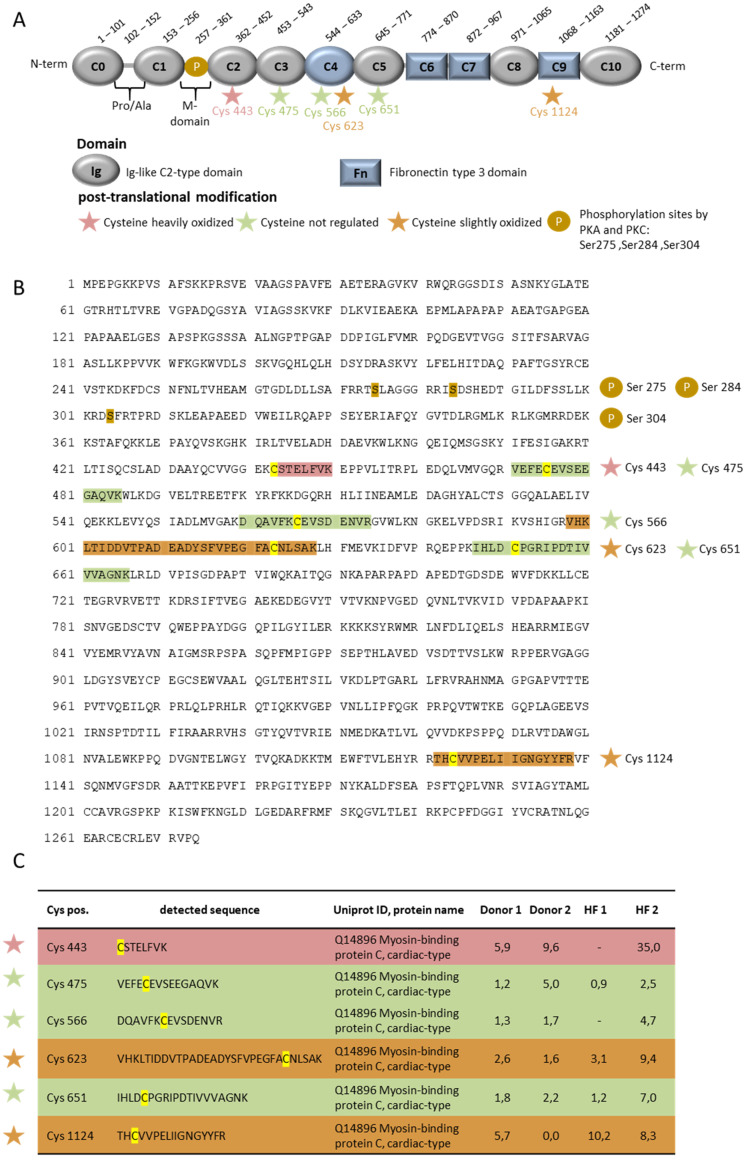
OxiCAT and mass spectrometry analysis of oxidized cysteine residues in cMyBP-C from myocardial biopsies of human HF patients. (**A**) Schematic representation of cMyBP-C illustrates the different domains. (**B**) Amino acid sequence of cMyBP-C with phosphorylation sites and the locations of the nonregulated and oxidized cysteine residues. (**C**) Oxidized and nonregulated cysteine residues of cMyBP-C with the representative mass spectrometric sequence UniProtKB-Q14896 (MYPC3_HUMAN), cardiac-type, updated: 2 December 2020. The numbers indicate the percentage of oxidation. *n* = 2 per group.

**Figure 5:**
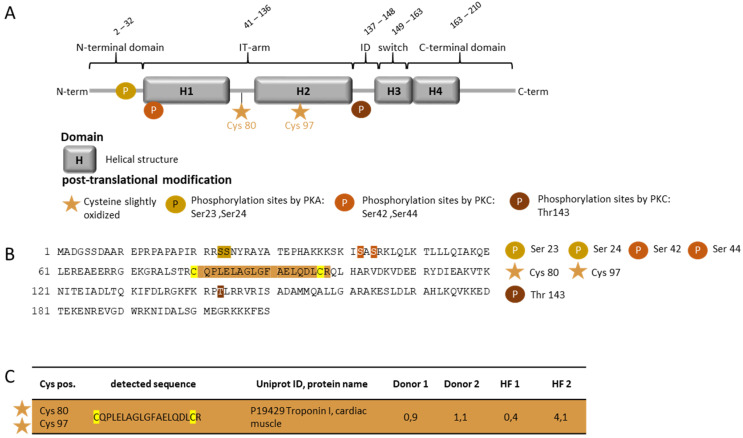
OxiCAT and mass spectrometry analysis of oxidized cysteine residues in cTnI from myocardial biopsies of human HF patients. (**A**) Schematic representation of cTnI illustrates the different domains. (**B**) Amino acid sequence of cTnI with PKA, PKD, and PKC phosphorylation sites and the location of the oxidized cysteine residue. (**C**) Oxidized and cysteine residue of cTnI with the representative mass spectrometric sequence UniProtKB—P19429 (TNNI3_HUMAN), cardiac muscle, updated: 2 December 2020. The numbers indicate the percentage of oxidation. *n* = 2 per group.

One heavily oxidized cysteine residue in the HF patient, cMyBP-C at position 443, and two slightly oxidized cysteine residues at 623 and 1124, were identified (UniProtKB- Q14896.4 (MYPC3_HUMAN) (Figure 4). Other cysteine residues, at positions 475, 566 and 651 in the sequence, were unregulated in the HF patients. The heavily oxidized Cys 443 is located at the Ig-like C2-type domain of cMyBP-C, and the two slightly oxidized residues in regions C4 and C9 (Figure 4). For cTnI, we could identify two slightly oxidized cysteines at positions 80 and 97 in one HF patient. Both the cysteine residues are located at the IT arm of cTnI (Figure 5). The IT arm is less mobile, and is responsible for the anchoring of the C-terminal domain of troponin C [34]. 

### 3.5. PKG Activity and Oxidation in End-Stage Human Failing Hearts

**Figure 6:**
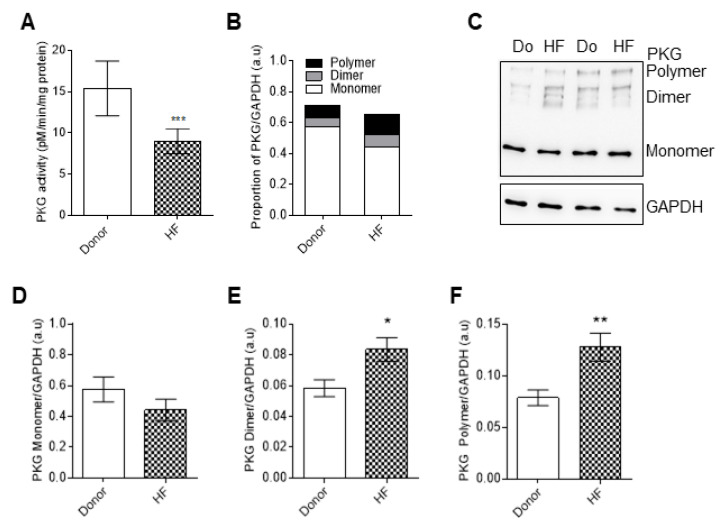
Protein kinase G (PKG) activity and analysis of monomer, dimer, and polymer formation in HF cardiomyocytes. (**A**) Protein kinase G (PKG) activity. (**B**) Proportion of PKG monomers, dimers, and polymers. (**C**) Representative blot of PKG monomers, dimers, and polymers. (**D**) PKG monomer levels. (**E**) PKG dimer levels. (**F**) PKG polymer levels. Data are shown as mean ± SEM. * *p* < 0.05/** *p* < 0.01/*** *p* < 0.001 donor vs. HF by Student *t*-test (*n* = 8–10, hearts per group).

As previously demonstrated by multiple studies, PKG-dependent hypo-phosphorylation of titin contributes to the increased stiffness that is observed in heart failure [23,35]. In our study, and due to increased oxidative stress, PKG activity was significantly decreased in HF compared to the donors (Figure 6A). Using Western blot analysis, PKG showed mono, dimer, and polymer formation (Figure 6B,C), suggesting PKG oxidation in end-stage human failing hearts, as shown by the increased levels of dimeric and polymeric formation (Figure 6D–F). 

### 3.6. The Functional Interplay between Ctni Oxidation and Phosphorylation in End-Stage Heart Failure

**Figure 7:**
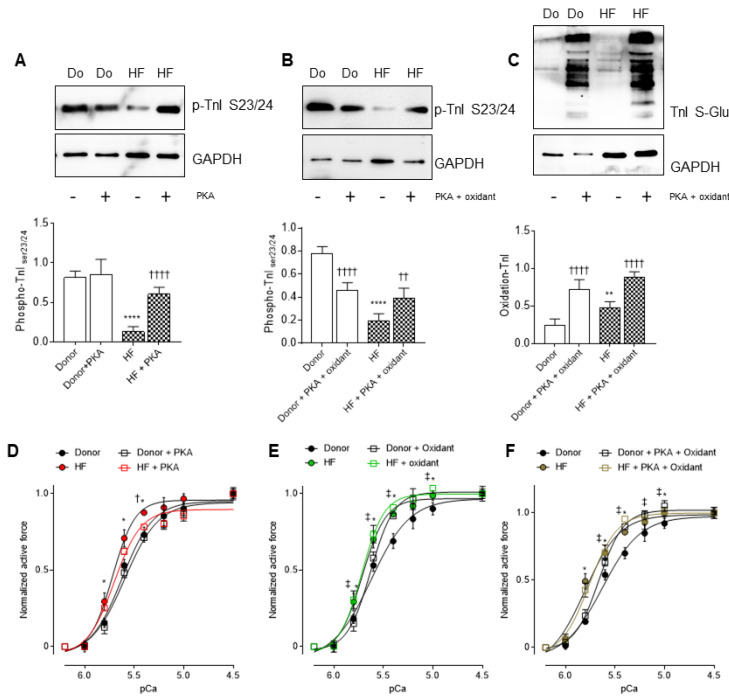
Effects of oxidative stress-induced hypophosphorylation on myocardial calcium sensitivity in HF. (**A**) Site-specific phosphorylation of cTnI 23/24 in the presence and absence of PKA. (**B**) Site-specific phosphorylation of cTnI 23/24 in the presence and absence of PKA + oxidant. (**C**) cTnI oxidation before and after treatment with PKA + oxidant (oxidized glutathione: GSSG). (**D**) Calcium sensitivity of donors and HF cardiomyocytes with and without PKA treatment at different calcium concentrations. (**E**) Calcium sensitivity of donors and HF cardiomyocytes with and without an oxidant supplementation at different calcium concentrations. (**F**) Calcium sensitivity of donors and HF cardiomyocytes before and after incubations with both PKA and an oxidant at different calcium concentrations. Curves are second-order polynomial fits to the means. Data are shown as mean ± SE (*n* = 16–20/4 cardiomyocytes/heart). * *p* < 0.05/** *p* < 0.01/**** *p* < 0.0001 donor vs. HF, † *p* < 0.05/†† *p* < 0.01/†††† *p* < 0.0001 HF before vs. after PKA + oxidant, ‡ *p* < 0.05 donor before vs. after PKA + oxidant by one-way ANOVA. *p*-values were corrected for multiple comparisons by the Tukey method.

Upon PKA treatment, cTnI phosphorylation at sites S23/S24 significantly increased in the HF tissues (Figure 7A). However, PKA administration, followed by an oxidant reagent GSSG, resulted in a significant increase in cTnI phosphorylation at S23/S24, in both the donor and HF tissues, but not to the level that was observed in the donors at baseline, or HF with PKA treatment (Figure 7B). The latter was accompanied by high cTnI oxidation levels (Figure 7C) before and after treatment with PKA plus the oxidant.

Next, we aimed to evaluate the contribution of oxidative stress to the phosphorylation-dependent alterations in the mechanical properties of HF myocytes. PKA treatment reduced the Ca^2+^ sensitivity of HF cardiomyocytes, nearly to the levels that have been measured in donor cardiomyocytes, whereas PKA had no effect on the Ca^2+^ sensitivity of the donor myocytes (Figure 7D). Both the donors and HF cardiomyocytes showed significant increases in Ca^2+^ sensitivity upon incubation with an oxidant reagent (Figure 7E). PKA administration, followed by the oxidant reagent, increased the Ca^2+^ sensitivity in the donor cardiomyocytes, and failed to restore the elevated Ca^2+^ sensitivity in the HF samples (Figure 7F), suggesting an oxidation-mediated hypo-phosphorylation of myofilament proteins, and a subsequent contractile dysfunction in HF cardiomyocytes. 

### 3.7. Pro-Inflammatory Cytokines and Oxidative Stress Parameters in End Stage Heart Failure Human Samples before and after Reduced Glutathione (GSH) Treatment

**Figure 8:**
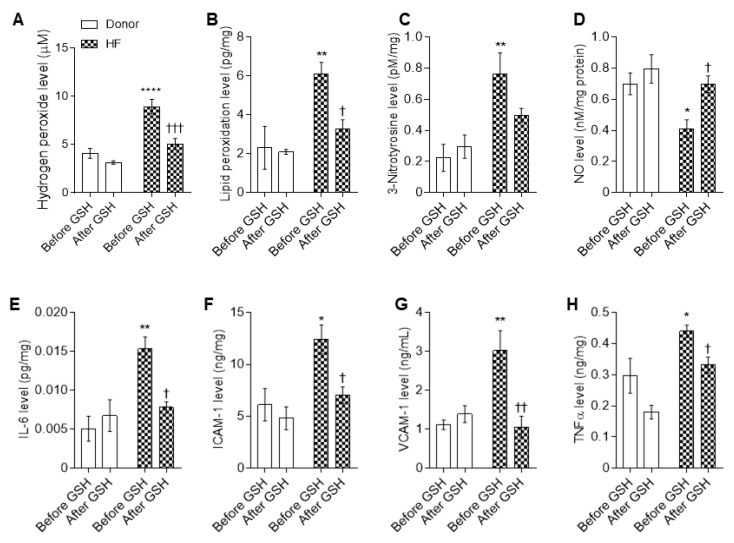
Proinflammatory cytokines and myocardial stress in HF cardiomyocytes. Concentration levels before and after reduced glutathione (GSH) treatment of (**A**) hydrogen peroxide (H_2_O_2_); (**B**) lipid peroxidation; (**C**) 3-nitrotyrosine; (**D**) nitric oxide (NO); (**E**) interleukin 6 (IL-6); (**F**) intercellular cell adhesion molecule-1 (ICAM-1); (**G**) vascular cell adhesion molecule-1 (VCAM-1); (**H**) tumor necrosis factor alpha (TNFα). Data are shown as mean ± SEM. * *p* < 0.05/** *p* < 0.001/**** *p* < 0.0001 donor baseline vs. HF baseline, † *p* < 0.05/†† *p* < 0.01/††† *p* < 0.001 before vs. after GSH. *n* = 8–10, hearts per group.

Oxidative stress markers, including hydrogen peroxide (H_2_O_2_), lipid peroxide (LPO), and 3-nitrotyrosine, were significantly elevated in human LV myocardial tissue compared to the donors (Figure 8A–C). We also detected high levels of pro-inflammatory cytokines, including interleukin-6 (IL-6), intercellular cell adhesion molecule-1 (ICAM-1), vascular cell adhesion molecule-1 (VCAM-1), and tumor necrosis factor alpha (TNFα), in the HF tissues compared to the donors (Figure 8E–H). Treatment with GSH decreased the elevated oxidative stress markers and pro-inflammatory cytokines to donor levels, whereas no effects were detected in the controls after GSH treatment (Figure 8A–C,E–H). Furthermore, we found nitric oxide (NO) bioavailability in HF patients to be significantly decreased compared to the donors (Figure 8D). GSH supplementation significantly increased the NO levels of the HF myocytes to donor levels, whereas (NO) levels were unchanged in the donor group upon GSH treatment (Figure 8D); this indicates oxidative stress-mediated attenuation of (NO) bioavailability in HF patients. 

### 3.8. Increased Myocardial Stiffness in HF Myocytes Is Reversed by PKA and GSH Treatment 

**Figure 9:**
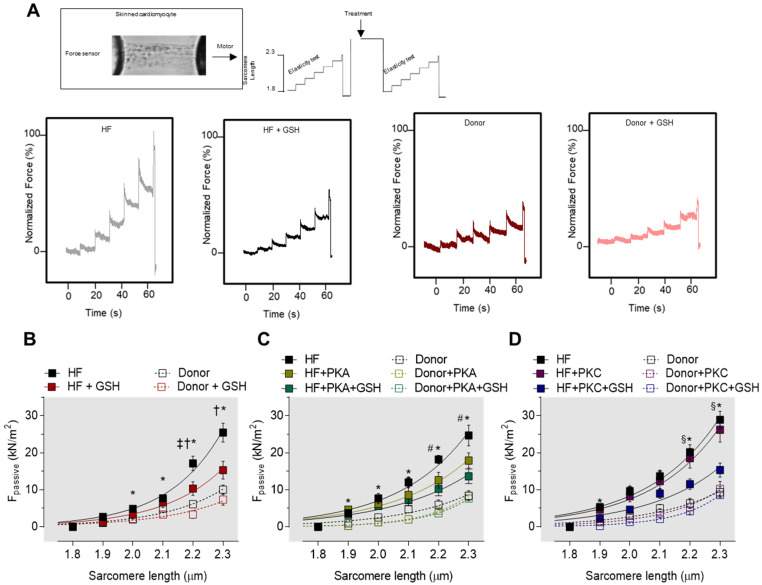
Cardiomyocyte passive stiffness of human HF myocardial biopsies. (**A**) Representative image of skinned cardiomyocyte and stepwise elasticity protocol. The panel below represents the original traces. (**B**) Passive stiffness of donors and HF cardiomyocytes before and after reduced glutathione (GSH) treatment at sarcomere length 1.8–2.3 µm. (**C**) Passive stiffness of donors and HF cardiomyocytes with and without protein kinase A (PKA) treatment and after incubations with both PKA and GSH at sarcomere length 1.8–2.3 µm. (**D**) Passive stiffness of donors and HF cardiomyocytes with and without PKC treatment and after incubations with both protein kinase C (PKC) and GSH at sarcomere length 1.8–2.3 µm. Curves are second-order polynomial fits to the means. Data are shown as mean ± SE (*n* = 16–20/4 cardiomyocytes/heart). * *p* < 0.05 donor vs. HF, † *p* < 0.05 HF before vs. after GSH or PKA, ‡ *p* < 0.05 donor before vs. after GSH, # *p* < 0.05 HF before vs. after PKA, and § *p* < 0.05 HF + PKA or +PKC vs. after HF + PKA + GSH or +PKC + GSH by one-way ANOVA. *p*-values were corrected for multiple comparisons by the Tukey method.

Cardiomyocyte Ca^2+^-independent passive force (F_passive_) was measured within a sarcomere length (SL) range between 1.8 and 2.3 μm. Figure 9A illustrates a representative elasticity test protocol and force recording for both the HF myocytes and donors. The passive SL–tension relationship of isolated skinned cardiomyocytes was generally steeper at SL 2.0 to 2.3 μm in the HF cardiomyocytes compared to the donor cardiomyocytes (Figure 9B). The treatment with GSH reduced the cardiomyocyte F_passive_ at SL 2.0 to 2.3 μm, while no effects of the GSH treatment were observed on the F_passive_ in cardiomyocytes from the donor group (Figure 9B). After PKA incubation, the F_passive_ of HF cardiomyocytes dropped significantly at SL 2.2 to 2.3 μm (Figure 9C), while it remained unchanged upon PKC treatment alone (Figure 9D). Supplementary PKA or PKC, followed by GSH treatment, resulted in a significant decrease in the HF myocytes F_passive_ at SL 2.2 to 2.3 μm (Figure 9C,D).

### 3.9. cTnI and cMyBP-C S-Glutathionylation in End Stage of Failing Heart Samples

Finally, we visualized cTnI and cMyBP-C S-glutathionylation using the Duolink proximity ligation assay (PLA). Confocal microscopic images of the immunostained HF tissue confirmed the S-glutathionylation of both cMyBP-C (Figure S1A,B) and cTnI (Figure S1C,D) in HF, indicating the direct oxidative stress effect on these myofilament proteins, and the potential contribution in contractile dysfunction.

## 4. Discussion

In this study we provide evidence for increased oxidative stress and inflammatory markers, with decreased NO and GSH levels, in human biopsies from end-stage heart failure patients. The changes were accompanied by hypoactivity of PKA and PKG, but hyperactivity of PKC. Changes in PKA and PKC activity were not associated with the kinase’s own oxidation; however, the drop in PKG activity was associated with enzyme oxidation in the HF tissues. Myofilamentary proteins, including cMyBP-C and cTnI, were both oxidized at the cysteine residues 443, 623 and 1124 (cMyBP-C), and 80 and 97 (cTnI). Of note, both proteins were hypo-phosphorylated in HF tissues. These modifications were accompanied by altered maximal tension and calcium sensitivity of force production, and passive stiffness, in demembranated single cardiomyocytes. All the functional parameters were restored by administration of the anti-oxidant GSH. Only passive stiffness was not completely reduced to the levels of the non-failing heart samples. Still, following GSH treatment, subsequent treatment of PKA or PKC improved the maximal tension and completely restored the calcium sensitivity of force production, while only PKA had an effect on passive stiffness.

### 4.1. Oxidation and Inflammation Parameters in End-Stage Heart Failure

Previous reports showed that increased levels of ROS associate with the development of multiple cardiovascular diseases, such as ischemia, coronary heart disease and atherosclerosis, hypertension, and HF via the oxidation of nucleic acids, proteins, or lipids [36,37,38,39,40,41]. Accordingly, in our current study, and by others, oxidative stress was markedly increased in end-stage HF patients, and was accompanied by significant changes in contractility, signaling pathways, [42], and protein modifications, including phosphorylation and S-glutahionylation of the myofilament proteins [13,14,43]. Increased oxidative stress could also be explained by increased pro-inflammatory cytokines, which may correlate with HF severity and mortality [1,44,45]. Mechanical stress, pressure and volume overload, potentially exacerbate inflammation in HF [7,46], as well as several non-cardiac comorbidities, such as obesity, diabetes mellitus, hypertension, and chronic obstructive pulmonary disease [13,47]. 

The combination of reduced levels of NO and glutathione (GSH), as seen in our data (as most abundant cellular antioxidant, and cytotoxic oxidative and inflammatory markers), strengthens the pivotal involvement of redox alterations in end-stage HF progression. The conclusion is strongly reinforced by our observation that the incubation of end-stage HF tissues with GSH restored the levels of all the inflammatory parameters, and the biomarkers for oxidative stress. 

### 4.2. Myofilament Proteins and Protein Kinases, Their Modification and Function under Oxidative Stress 

The altered maximum Ca^2+^-activated tension and Ca^2+^ sensitization that was observed in HF cardiomyocytes is mainly caused by both the hypo-phosphorylation of cTnI and cMyBP-C, and the S-glutathionylation of cMyBP-C, but not cTnI. Our data showed elevated levels of ROS/RNS, which may contribute to the S-glutahionylation of cMyBP-C, cTnI, and the N2B isoform of titin. We found S-glutahionylated cysteine residues of TnI only at the IT arm of TnI, which essentially has no functional effect on TnI, so it could not contribute the mechanical alterations that were measured in the HF cardiomyocytes. S-glutathionylation of troponin I has an effect on the movement of troponin I towards the troponin C bound state, resulting in increased interactions between troponin I and troponin C at lower Ca^2+^ levels that can potentially reduce the inhibitory action of TnI on actin [48,49,50]. The SH groups of troponin subunits, in a reduced state, play a key role in producing and maintaining the troponin complex. Oxidized troponin, however, has only a minor effect on the Ca^2+^ sensitivity of actomyosin ATPase, which is reversible upon dithiothreitol administration [48]. As a consequence, oxidized troponin I does not bind to troponin T [48,49,50]. Although troponin I harbors several cysteine residues, some of these cysteines are insensitive to Ca^2+^-induced conformational changes in native troponin I [51], which is in line with our current findings. 

Further, cMyBP-C is S-glutathionylated at Cys479, Cys627, and Cys655 of the C3, C4, and C5 domains in the mouse model [24,52,53]. The S-glutathionylation sites associate with the impairment of cellular relaxation, with unaltered cellular Ca^2+^ dynamics. The reduction in cMyBP-C S-glutathionylation ameliorates diastolic dysfunction [52], suggesting a strong correlation between S-glutathionylation and diastolic dysfunction. S-glutathionylation of cMyBP-C increases myofilament Ca^2+^ sensitivity, suggesting the contribution of cMyBP-C S-glutathionylation to the maintenance of longitudinal rigidity and cross-bridge kinetics [53], and may explain the mechanical deterioration that was observed in the cardiomyocytes from HF patients. 

In addition to the mechanical contribution of cMyBP-C glutathionylation to altered force production, oxidative stress-related redox modification of titin, disulfide bonding in the cardiac-specific titin N2-Bus decreases the extensibility of N2B, stiffens the whole titin molecule, and increases cardiomyocyte passive tension [23]. It has been shown that the internal disulfide bridge in titin N2-Bus may interfere with the (de)phosphorylation of PKG-dependent stiffness, and influence ligand binding [54], mechanosensing, and protein quality control [28].

In addition, titin S-glutathionylation in the Ig-domains of the elastic I-band region inhibits the folding, and weakens the refolding, of domains [17], thereby reducing titin-based stiffness. Our study suggests that the oxidation of cMyBP-C and titin all contribute to the altered maximal tension and calcium sensitivity of force production and passive stiffness in end-stage HF patients. 

### 4.3. Interplay between Myofilament Proteins Oxidation and Phosphorylation under Oxidative Stress 

Oxidation may additionally influence other post-translational modifications and their interplay [55], which represents a complex regulatory network and a broad system that regulates the cardiomyocyte function. Oxidative stress increases phosphorylation by stimulating protein kinases and/or inhibiting protein phosphatases [56]. A redox-sensitive cysteine, found in protein tyrosine phosphatases, is highly predisposed to ROS-dependent inactivation, resulting in increased protein tyrosine phosphorylation. In addition to the oxidation of cMyBP-C and cTnI, both the proteins can be also phosphorylated by PKA and PKC, to fine-tune contractility [57] and contribute to myocardial stiffness. Myocardial stiffness is, however, mainly regulated by titin and its phosphorylation by PKG [18,32,58]. We found hypoactivity of PKG in the HF samples, which has been described formerly in HF patients with a preserved ejection fraction [13,14,33,43]. PKG hypoactivity may be explained by the reduced NO levels that have been observed in HFpEF patients [13,14,33,43]. Lower NO bioavailability results in less cGMP production, and thus in reduced activation of PKG. Reduced PKG activity has been related to a higher passive stiffness, due to the hypo-phosphorylation of titin and increased oxidative stress [13,14,33,43]. Moreover, under oxidative stress, oxidative modifications, such as disulfide bonding or glutathionylation of the enzymes, may occur, which may uncouple their activity from cAMP or cGMP [59]. We indeed observed prominent PKG oxidation in the HF tissues. Dimer and polymer formation is a hallmark for oxidized PKA, PKC, and PKG [59]. Here, however, PKG oxidation correlated with PKG hypoactivity, which may explain the hypo-phosphorylation of titin. In addition, PKG–titin interactions, which are potentially impaired due to the S-glutathionylation of titin, could also contribute to the hypo-phosphorylation of titin, and thereby also modulate the cardiomyocyte stiffness in these patients. Together, this demonstrates that pleiotropic factors determine PKG hypoactivity and the hypo-phosphorylation of titin, while increasing passive stiffness [33,43]. 

Additionally, PKA activity is also found to be reduced in end-stage HF patients, whereas PKC hyperactivity is observed, which is a phenomenon that has been previously described [60,61], and may contribute to the contractile dysfunction observed in our patients.

In the healthy heart, PKA-dependent phosphorylation of cTnI decreases the Ca^2+^ sensitivity of the actin–myosin interaction, and fastens relaxation as a response to β-adrenergic stimulation. PKA-phosphorylated cMyBP-C and phospholamban, which is the regulator of the sarcoplasmic reticulum Ca^2+^ ATPase, operate in concert with phosphorylated cTnI. This cooperation is facilitated by the direct interaction of cMyBP-C with cTnI [62]. PKC phosphorylation of cTnI or cMyBP-C normally balances the PKA phosphorylation effects [63], and has widely converse effects, which, however, might depend on the PKC isoform involved [64]. 

The reduced PKA activity, despite the elevated circulating catecholamines in HF, can be explained by the desensitization of β_1_ adrenoceptors, and blunted cAMP production by receptor-coupled adenylyl cyclases and enhanced activities of phosphodiesterases. PKA and PKC are likely non-oxidized in these human failing biopsies, as seen from our data, indicating that oxidation does not contribute directly to the observed alterations in PKA and PKC activity. The decreased PKA and increased PKC activity imply that PKA-dependent phosphorylation of cMyBP-C and cTnI should be decreased, whereas PKC-dependent phosphorylation of both the proteins should be increased. Nevertheless, in our study, both the proteins were hypo-phosphorylated. This can be explained by the fact that cMyBP-C contains several phosphorylation sites, whereby S273, S282 and S302 (mouse sequence) are phosphorylated by PKA, while PKC phosphorylates only S282 and S302 [65,66]. In our current study, we found decreased phosphorylation at cTnI PKA sites S23/S24 and at PKC site S42 (human canonical sequence), but the PKC site T143 was unaltered, despite the increased PKC activity. There are the following several explanations for this apparent discrepancy of cTnI hypo-phosphorylation, despite the increase in PKC activity: (1) oxidized cTnI might exhibit an altered affinity to PKC, by altering the structural and dynamic properties, which is supported by the location of the oxidized cysteine residues; (2) PKC sites could be phosphorylated by another protein kinase that is not yet known to the archetypical PKA/PKC sites in cTnI, and if there is any imbalance of these kinases then the one may cancel the other, thereby affecting the phosphorylation status of the site. (3) other phosphorylated sites might inhibit phosphorylation by PKC at S42 (human canonical sequence) in cTnI. Such an interconnection between different intramolecular phosphorylation sites has been described for cTnI [67,68]. 

To better understand the changes in the PKA-dependent phosphorylation of myofilament proteins, we investigated whether cTnI hypo-phosphorylation was attributable to the oxidative stress that was observed in the HF patients. Upon PKA treatment, site-specific phosphorylation of cTnI S23/S24 was increased in the HF tissues. However, PKA administration, followed by an oxidant reagent, resulted in a decrease in cTnI S23/S24 phosphorylation in both the donor and HF tissues, to lower levels compared to the non-treated tissues. The latter was accompanied by high cTnI oxidation levels, indicating that protein oxidation mediates the dysregulated phosphorylation in both HF and donor hearts, suggesting altered PKA affinity and/or the accessibility to phospho-sites neighboring the oxidized cysteines.

Additionally, we evaluated the contribution of oxidative stress to the phosphorylation-dependent alterations in the mechanical properties of HF myocytes. PKA administration in vitro reduced the elevated Ca^2+^ sensitivity of HF cardiomyocytes to the level observed in donor cardiomyocytes, whereas both the donors and HF cardiomyocytes showed significant increases in Ca^2+^ sensitivity upon treatment with an oxidant reagent. PKA administration, followed by the oxidant reagent, increased the Ca^2+^ sensitivity in the donor cardiomyocytes, and failed to restore the elevated Ca^2+^ sensitivity in the HF samples, suggesting oxidation-mediated hypo-phosphorylation of myofilament proteins, and a subsequent contractile dysfunction in HF cardiomyocytes. 

## 5. Conclusions and Therapeutic Implications

While in the healthy heart oxidative modifications and antioxidant defense systems are balanced and inflammatory processes are low, in the end-stage failing heart, oxidative stress and inflammation are prominent, causing oxidation of myofilament proteins, dysregulated phosphorylation, and contractile dysfunction. A result of both the direct and indirect effects of oxidative stress on proteins, either through oxidation or via signaling pathways that are involved in protein phosphorylation, and the interplay between these two pathways, represents a complex regulatory network and a broad cell regulatory system, and the respective influence of these modifications on proteins function and thereby on cellular function. Hence, targeting inflammation and oxidative stress appears to be a valid therapeutic approach in HF, and may influence the (in)direct effects of oxidative stress. Furthermore, providing a combination of active protein kinases and antioxidants should ameliorate the alterations that occurred in end-stage HF, and reverse the detrimental effects on contractility. Notably, PKA or PKC treatment could restore contractility alterations when combined with GSH. 

There are several therapeutic options to treat oxidative stress-associated cardiovascular diseases. Antioxidants, such as nutritional supplements, demonstrated therapeutic benefits in vitro; however, clinical trials reported the failure of non-specific vitamin supplementation in improving cardiac function [69]. Astaxanthin demonstrated beneficial effects in thrombotic disease [70,71]. Activators of the antioxidant defense system, such as NRF2 activators, have been shown to be protective in I/R injury and atherosclerosis [72]. CXL-1427, an HNO donor, was used to uncouple NOS in HFrEF [73]. A recent study by us provided evidence on the beneficial effects of empagliflozin in HF. We found that empagliflozin improved endothelial and cardiomyocyte function through reducing oxidative stress and restoring the diminished NO–sGC–cGMP–PKG pathway in HFpEF [13,74]. Furthermore, targeting the altered stress-signaling pathways and their downstream effectors might protect from proteotoxicity and cardiac remodeling in HF [75].

In addition to pharmacological interventions, treatment of comorbidities and reducing risk factors are essential in the management of chronic inflammation and oxidative stress associated with HF. Taken together, our results suggest that antioxidant treatment combined with active kinases, especially PKA, might restore the cardiomyocyte failure in HF.

## Figures and Tables

**Table 1:** List of antibodies used for Western blots and imaging.

Antibody	Company	Product Number	Buffer	Dilution
Cardiac Troponin I antibody	Abcam	ab 47003	BSA-TBST	1:1000
Phospho-Troponin I (Cardiac) (S23/S24) antibody	Cell Signaling Technology	4004S	BSA-TBST	1:1000
Cardiac Troponin I (phospho S43) antibody/mouse canonical sequence	Abcam	ab 196005	BSA-TBST	1:1000
Cardiac Troponin I (phospho T143) antibody	Abcam	ab 58546	BSA-TBST	1:1000
PKA C-α antibody	Cell Signaling	4782	BSA-TBST	1:1000
PKCα antibody	Cell Signaling	2056	BSA-TBST	1:1000
PKG antibody	Cell Signaling	13511	BSA-TBST	1:1000
Cardiac myosin binding protein C (phospho S282) antibody	Enzolife sciences	ALX-215-057-R050	BSA-TBST	1:500
Glutathione	Abcam	ab19534	BSA-TBST	1:200
Total cardiac myosin binding protein C antibody	Sigma-Aldrich	HPA040147	BSA-TBST	1:500

## Data Availability

Data is contained within the article.

## Supplementary Materials

The following are available online at https://www.mdpi.com/article/10.3390/antiox10071134/s1, Figure S1: A. Representative immunofluorescence confocal images (maximum intensity projection of z-stacks) of cardiomyocytes stained with DAPI (blue), WGA (red), and Duolink in Situ detection of GSH and cMyBP-C interaction (green). B. Representative images of Duolink in Situ detection of GSH and cMyBP-C interaction in a z-stack. C. Representative immunofluorescence confocal images (maximum intensity projection of z-stacks) of cardiomyocytes stained with DAPI (blue), WGA (red), and Duolink in Situ detection of GSH and cTnI interaction (green). D. Representative images of Duolink in Situ detection of GSH and cTnI interaction in a z-stack.
