# Supplementary material for: The Interplay between S-Glutathionylation and Phosphorylation of Cardiac Troponin I and Myosin Binding Protein C in End-Stage Human Failing Hearts
Source: Antioxidants (Basel). 2021 Jul 16;10(7):1134. doi: 10.3390/antiox10071134 (PMC8301081; doi:10.3390/antiox10071134)
Supplement: Supplementary file 1 [file antioxidants-10-01134-s001.zip › antioxidants-1260909-supplementary.pdf]

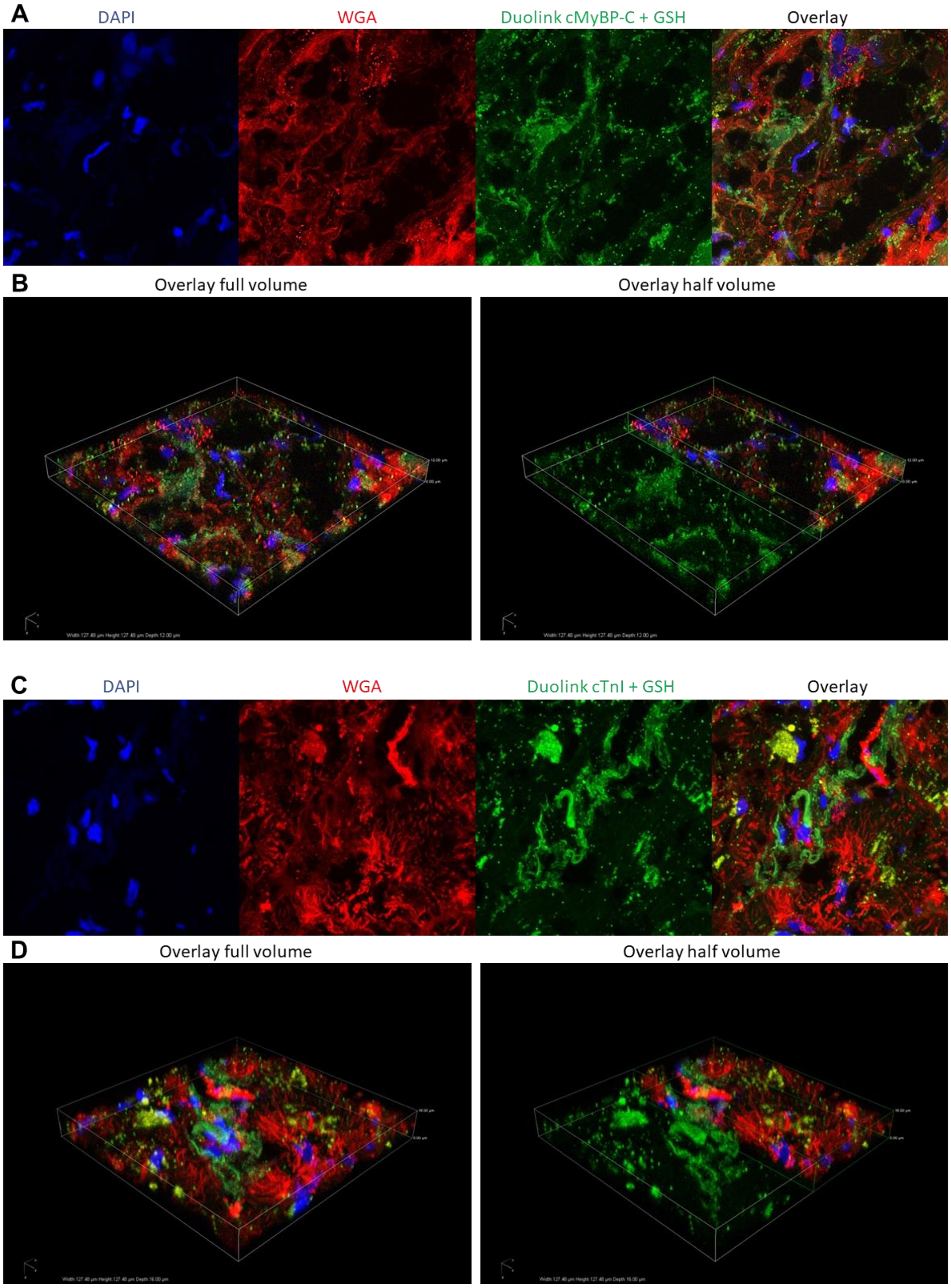

Figure S1. cMyBP-C and cTnI S-glutathionylathion in HF tissues A. Representative immunofluorescence confocal images (maximum intensity projection of z-stacks) of cardiomyocytes stained with DAPI (blue), WGA (red), and Duolink in Situ detection of GSH and cMyBP-C interaction (green). **B.** Representative images of Duolink in Situ detection of GSH and cMyBP-C interaction in a z-stack. **C**. Representative immunofluorescence confocal images (maximum intensity projection of z-stacks) of cardiomyocytes stained with DAPI (blue), WGA (red), and Duolink *in Situ* detection of GSH and cTnI interaction (green). **D.** Representative images of Duolink in Situ detection of GSH and cTnI interaction in a z-stack.
